# Melatonin Reverses the Loss of Stemness Induced by TNF-*α* in Human Bone Marrow Mesenchymal Stem Cells through Upregulation of YAP Expression

**DOI:** 10.1155/2019/6568394

**Published:** 2019-12-30

**Authors:** Xudong Wang, Tongzhou Liang, Jincheng Qiu, Xianjian Qiu, Bo Gao, Wenjie Gao, Chengjie Lian, Taiqiu Chen, Yuanxin Zhu, Anjing Liang, Peiqiang Su, Yan Peng, Dongsheng Huang

**Affiliations:** ^1^Department of Orthopedics, Sun Yat-sen Memorial Hospital of Sun Yat-sen University, Guangzhou, Guangdong 510120, China; ^2^Department of Orthopedics, The First Affiliated Hospital of Sun Yat-sen University, Guangzhou, Guangdong 510080, China

## Abstract

Mesenchymal stem cells (MSCs) are promising candidates for tissue regeneration and disease treatment. However, long-term *in vitro* culture results in loss of MSC stemness. The inflammation that occurs at stem cell transplant sites (such as that resulting from TNF-*α*) is a contributing factor for stem cell treatment failure. Currently, there is little evidence regarding the protective role of melatonin with regard to the negative effects of TNF-*α* on the stemness of MSCs. In this study, we report a melatonin-based method to reduce the inflammatory effects on the stemness of bone marrow mesenchymal stem cells (BMMSCs). The results of colony formation assays, Alizarin red staining, western blotting, and reverse transcription-polymerase chain reactions suggest that melatonin can reverse the inflammatory damage caused by TNF-*α* treatment in the third, seventh, and tenth generations of primary BMMSCs (vs. control and the TNF-*α*-treated group). Meanwhile, a detailed analysis of the molecular mechanisms showed that the melatonin receptor and YAP signaling pathway are closely related to the role that melatonin plays in negative inflammatory effects against BMMSCs. In addition, *in vivo* experiments showed that melatonin could reverse the damage caused by TNF-*α* on bone regeneration by BMMSCs in nude mice. Overall, our results suggest that melatonin can reverse the loss of stemness caused by inflammatory factor TNF-*α* in BMMSCs. Our results also provide a practical strategy for the application of BMMSCs in tissue engineering and cell therapy.

## 1. Introduction

Bone marrow mesenchymal stem cells (BMMSCs) are spindle-shaped adherent cells that were originally detected in bone marrow cultures. Subsequent studies have suggested that MSCs are mainly present in the bone marrow stroma [1]. Stemness is defined as the ability of stem cells to maintain the potential for proliferation and multiple routes of differentiation [2]. Under different induction conditions, MSCs can differentiate into a variety of mesodermal tissue cells, such as chondrocytes, osteoblasts, cardiomyocytes, and adipocytes. Meanwhile, MSCs can also differentiate into endoderm and ectoderm cells, such as hepatocyte-like cells and neuron-like cells [3]. In addition, multiple advantages of using MSCs in clinical applications have been reported, including low immunogenicity, multidirectional differentiation potential, induction of immune tolerance, immunosuppression, and lack of associated ethical issues [4]. Therefore, MSCs have become a primary candidate for cell therapy and tissue engineering. However, in clinical practice, MSCs need to be expanded *in vitro* to obtain sufficient quantities, but this subjects them to the deleterious effects of replicative aging. In addition, MSCs that experience oxidative stress may undergo premature aging, which can significantly affect their ability to differentiate into different types of cells [5, 6]. These factors limit the clinical application of MSCs [7].

Tumor necrosis factor-*α* (TNF-*α*) is a cytokine produced following the activation of macrophages/monocytes. This factor plays an important role in the maintenance of cell stemness and in fat differentiation [8]. For example, TNF-*α* can inhibit the differentiation of stem cells into osteoblasts through multiple signaling pathways, including through wingless-type MMTV integration site family members (Wnt), bone morphogenetic protein- (BMP-) Smads, mitogen-activated protein kinase (MAPK), and nuclear transcription factor kappa B (NF-*κ*B) signaling [9, 10]. However, it is unclear what associated factors are required for TNF-*α* to maintain stemness and the potential for cell differentiation in BMMSCs [11].

Melatonin is a hormone secreted mainly from the pineal gland that has proven to have widespread effects [12]. Previous studies have shown that melatonin regulates various physiological functions such as sleep, circadian rhythms, and neuroendocrine activities [13]. Melatonin has well-known antioxidant properties and has been shown to eliminate excessive free radicals and increase synthesis of intracellular antioxidant enzymes [14, 15]. Melatonin protects cells from proinflammatory cytokines by reducing active oxygen production and increasing superoxide dismutase production [16]. In 1999, Dun et al. confirmed that high levels of melatonin were present in the bone marrow [17]. In recent years, melatonin has been shown to regulate pluripotent differentiation of MSCs [18]. Radio et al. found that melatonin enhanced alkaline phosphatase activity in human MSCs via the MAPK signaling pathway. Moreover, *in vivo* studies have confirmed that melatonin promotes bone formation in mice at pharmacological concentrations [19]. However, there is still little evidence as to how melatonin reverses the inhibition of stemness by TNF-*α* in MSCs. Therefore, the detailed roles of melatonin and TNF-*α* in the stemness of MSCs deserve further investigation.

In this study, we employed TNF-*α* to simulate inflammation in the environment of the third, seventh, and tenth generations of BMMSCs. Colony formation, Alizarin red staining, western blotting, and RT-PCR were used to assess the molecular effects of TNF-*α*- and/or melatonin-treated human BMMSCs. In addition, an osteoporosis nude mouse model was used to study pathological changes with hematoxylin and eosin (H&E) staining and a micro-CT approach. The results obtained in this study provide detailed information regarding BMMSC stemness that will be useful in future clinical applications.

## 2. Materials and Methods

### 2.1. Isolation and Culture of Human BMMSCs

These experiments were approved by the Ethics Committee of Sun Yat-sen University. The MSCs were donated by three healthy volunteers. All participants fully understood the experimental procedures and provided written informed consent. Human BMMSCs were isolated as described previously [20–23]. Briefly, human bone marrow (8–10 mL) was isolated and the cells were counted and plated in 75 cm^2^ flasks at a density of 2 × 10^5^/cm^2^ in DMEM (Invitrogen, USA) with 20% fetal bovine serum. Nonadherent cells were removed after 24 h. Subsequently, the media were changed every 3 days. When the cells reached 80%–90% confluence, the BMMSCs were expanded to passage 2 using human recombinant trypsin (Invitrogen, USA). Cells at passage 3 (P3), passage 7 (P7), and passage 10 (P10) were harvested for future analyses.

### 2.2. Flow Cytometry

BMMSCs were analyzed by flow cytometry (DxFLEX, Beckman Coulter, USA). Cells at P3 were incubated in PE anti-human CD73 (Ecto-5′-nucleotidase) (BioLegend, USA), PE anti-human CD73 (Ecto-5′-nucleotidase) (BioLegend, USA), APC anti-human CD90 (Thy1) (BioLegend, USA), APC anti-human CD34 (BioLegend, USA), Alexa Fluor® 488 anti-human CD45 (BioLegend, USA), and Alexa Fluor® 488 anti-human CD105 (Abcam, USA) at concentrations specified by the manufacturer. Corresponding isotype-identical antibodies served as controls.

### 2.3. BMMSC Treatment

BMMSCs at P3, P7, and P10 were induced toward osteogenic differentiation and adipogenic differentiation. For osteogenic induction, BMMSCs were plated in DMEM supplement with osteogenic differentiation assay (LiuheBio, China). After 2 weeks, differentiated BMMSCs were stained with Alizarin red S (ARS) solution (Sigma-Aldrich, USA). The detailed experimental processes of the tests were conducted as described previously [24]. For adipogenic induction, BMMSCs were seeded in mesenchymal stem cell-adipocyte differentiation medium (ScienCell, China). The experimental protocol was followed as provided by the manufacturer. The colony formation assays were conducted with untreated BMMSCs and BMMSCs treated with 20 ng/mL TNF-*α*, TNF-*α*+100 *μ*M melatonin (Mel), TNF-*α*+Mel+10 *μ*M luzindole (Luz), or TNF-*α*+Mel+5 *μ*M verteporfin (VP, Yes-associated protein (YAP) inhibitor). About 500 cells per well were added to six-well culture plates. After a 2-week incubation, the cells were washed twice with PBS and stained with toluidine blue solution (BestBio, China). The clone formation efficiency was calculated as [number of colonies]/[number of cells inoculated]. Melatonin, TNF-*α*, Luz, and VP were obtained from Sigma-Aldrich (USA).

### 2.4. RT-PCR

RNAiso Plus reagent (Roche, Switzerland) was used to exact total RNA from BMMSCs. Reverse transcription-PCR (RT-PCR) analysis was carried out in 20 *μ*L final volume from 400 ng total RNA using the TaKaRa PrimeScript II 1st Strand cDNA Synthesis Kit (D6210A, TaKaRa, Japan) according to manufacturer's instructions. *GAPDH*, *RUNX2*, *OPN*, *OCN*, *HTERT*, *SOX2*, *C-MYC*, *NANOG*, *PPARγ-2*, *LPL*, *ADIPONECTIN*, and *YAP* mRNA levels were determined by RT-PCR, and primers were utilized as described in Table 1. Reactions were performed in 20 *μ*L volumes containing 2x SYBR® *Premix Ex Taq*™ (TaKaRa, Japan), PCR Forward Primer (10 *μ*M), PCR Reverse Primer (10 *μ*M), cDNA template, and ddH_2_O. RT-PCR was performed using a Bio-Rad CFX96 Real-Time PCR System. Thermal cycling conditions were as follows: 10 min at 95°C, followed by 40 cycles of 95°C for 10 s, 60°C for 20 s, and 72°C for 30 s. Data were collected and calculated using the Bio-Rad CFX Manager Software1.6. RNA expression was calculated based on a relative standard curve with the 2^−*ΔΔ*ct^ method.

### 2.5. Western Blotting

Total cellular protein from differently treated BMMSCs was isolated by the addition of 1% PMSF and RIPA lysis buffer (50 mM Tris-HCl (pH 7.4), 150 mM NaCl, 1% NP-40, 0.1% SDS). After boiling in SDS-PAGE sample buffer for 5 min, the samples underwent sodium dodecylsulfate-polyacrylamide gel electrophoresis (SDS-PAGE). The proteins were then transferred onto polyvinylidene difluoride (PVDF) membranes (Millipore, USA). After being blocked for 1 h at room temperature, the membranes were incubated with a 1 : 1000 dilution of the following antibodies (Abs) from Abcam (Cambridge, UK) overnight: Abs against HTERT, SOX2, C-MYC, NANOG, OPN, OCN, RUNX2, PPAR*γ*-2, Lipoprotein lipase (LPL), ADIPONECTIN, and GAPDH. Before detection with an ECL chemiluminescence detection kit (Beyotime Institute of Biotechnology, China), proteins were incubated with horseradish peroxidase- (HRP-) conjugated goat anti-rabbit IgG (H&L) and HRP-conjugated goat anti-mouse IgG (H&L) secondary antibodies (1 : 5000 dilution) for 1 h at room temperature (Thermo Scientific, USA). The bands were examined with ImageJ software (National Institutes of Health, USA).

### 2.6. Animal Treatment

Eight-week-old Balb/c nude mice were obtained from Charles River (Beijing, China). Sixty nude mice were randomly divided into six groups, including control (Con, *n* = 10), osteoporosis simulation group (OVX, *n* = 10), OVX+P3 BMSC treatment (OVX+P3, *n* = 10), OVX+P3 BMSCs+20 ng/mL TNF-*α* treatment (OVX+P3+TNF-*α*, *n* = 10), OVX+P3 BMSCs+TNF-*α*+100 *μ*M Mel treatment (OVX+P3+TNF-*α*+Mel, *n* = 10), and OVX+P3 BMSCs+TNF-*α*+Mel+5 *μ*M verteporfin (OVX+P3+TNF-*α*+Mel+VP, *n* = 10). After the model of osteoporosis was established (ovary removal surgery), BMMSCs treated with Mel, TNF-*α*, and VP were injected into the tail veins. Eight weeks after modeling, femur tissues were collected for further experimentation.

### 2.7. H&E Staining

H&E staining was conducted as previously reported [25]. Bone samples were fixed in 10% buffered formalin and embedded in paraffin. Three- to five-micrometer thick sections were stained with hematoxylin (Sigma H 3136) for 10 min and with eosin (Sigma E 4382) for 1 min to establish the diagnosis areas.

### 2.8. Bone Microstructure Observation

To observe the bone microstructure, micro-CT examination was carried out with SCANCO Medical AG (Switzerland) before sacrificing, as previously described [26]. The parameters were set as follows: voltage = 50 kV; current = 500 *μ*A; resolution = 8.96 *μ*m; exposure time = 5 ms; scanning layer = 2394. Subsequently, the microstructure parameters of bone, such as bone volume/total volume (BV/TV), trabecular number (Tb.N), trabecular thickness (Tb.Th), trabecular separation (Tb.Sp), and trabecular pattern factor, were measured automatically using the Siemens Preclinical Imaging System in a multislice in standard resolution mode [27, 28].

### 2.9. Statistical Analysis

Statistical differences in this study were assessed using SPSS 18.0 software. The mean values of the statistical results for the measured data are presented as the means ± standard error. The values in this study were analyzed by Student's *t*-test. *P* values < 0.05 were considered to be a significant difference between two measurements.

## 3. Results

### 3.1. BMMSC Stemness Was Decreased along with Cell Passage

**Figure 1:**
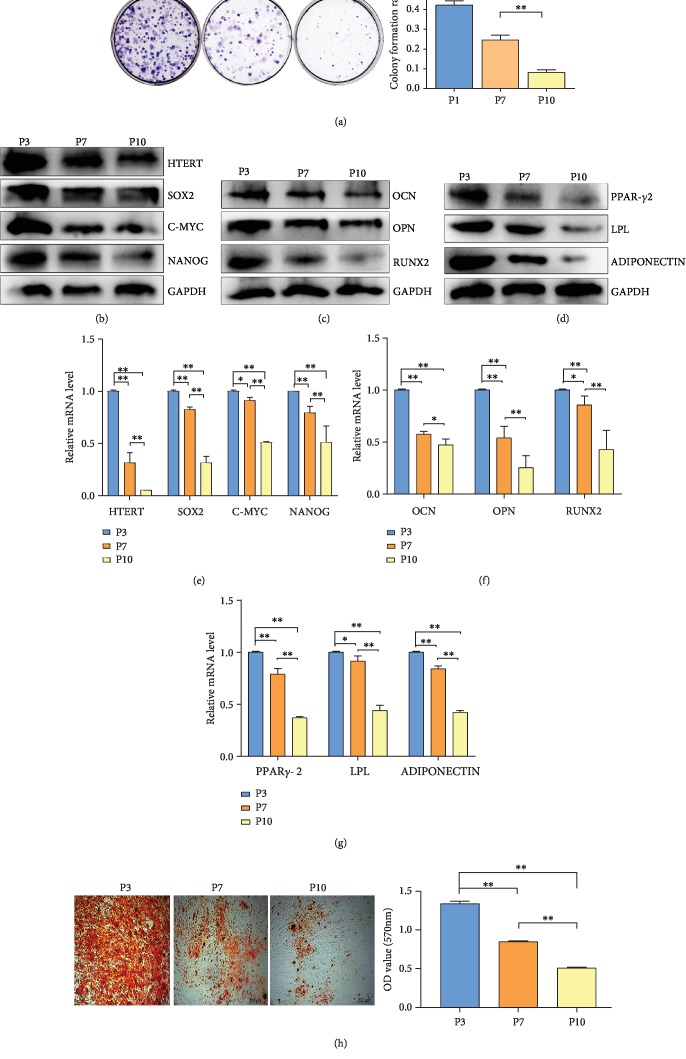
HMSC stemness assay. (a) Colony formation assays of HMSCs. Quantitative analysis of colony formation ratio of HMSCs. (b) Western blot analysis of biomarkers of HMSC stemness (HTERT, SOX2, C-MYC, and NANOG). (c) Western blot analysis of biomarkers of osteogenic differentiation of HMSCs (OPN, OCN, and RUNX2). (d) Western blot analysis of biomarkers of adipose differentiation of HMSCs (PPAR*γ*-2, LPL, and ADIPONECTIN). (e) RT-PCR analysis of biomarkers of HMSC stemness. (f) RT-PCR analysis of biomarkers of osteogenic differentiation of HMSCs. (g) RT-PCR analysis of biomarkers of adipose differentiation of HMSCs. (h) Alizarin red staining and quantification examination of osteogenic differentiation of HMSCs. P3: primary cells expanded to the third generation; P7: primary cells expanded to the seventh generation; P10: primary cells expanded to the tenth generation. ^∗^*P* < 0.05, ^∗∗^*P* < 0.01.

In this study, BMMSCs at P3 were subjected to flow cytometric analysis to estimate the presence of surface markers (Supplementary [Supplementary-material supplementary-material-1]). Most BMMSCs (>95%) were positive for CD73, CD90, and CD105, whereas <2% were positive for CD45 or CD34. The relationship between the stemness of BMMSCs and cell inheritance was examined by colony formation assays, western blotting, RT-PCR, and Alizarin red staining. For colony formation analysis, we selected the primary BMMSCs at P3, P7, and P10. The results showed that colony formation of P7 and P10 BMMSCs was significantly reduced compared with that of P3 and primary BMMSCs (*P* < 0.01) (Figure 1(a)). For western blot analysis, we evaluated biomarkers of BMMSC stemness (HTERT, SOX2, C-MYC, and NANOG), osteogenic differentiation (RUNX2, OPN, and OCN), and adipose differentiation (PPAR*γ*-2, LPL, and ADIPONECTIN) in P3, P7, and P10 BMMSCs (Figures 1(b)–1(d)). The results revealed that abundance of proteins reflecting BMMSC stemness, osteogenic differentiation, and adipose differentiation was significantly downregulated in P7 and P10 cells compared with P3 cells. Meanwhile, RT-PCR analyses gave similar results for these biomarkers as western blot analyses. mRNA abundances of these biomarkers in P7 and P10 cells were all significantly reduced compared with those in P3 cells (*P* < 0.01) (Figures 1(e)–1(g)). In addition, we employed Alizarin red staining to investigate osteogenic differentiation in P3, P7, and P10 BMMSCs. Quantification analysis revealed that the optical density (OD) of staining in P7 and P10 cells was lower than that in P3 cells (Figure 1(h)). In summary, stemness was closely correlated with the generation of BMMSCs observed.

### 3.2. Melatonin Can Reverse the Loss of BMMSC Stemness Caused by an Inflammatory Environment

**Figure 2:**
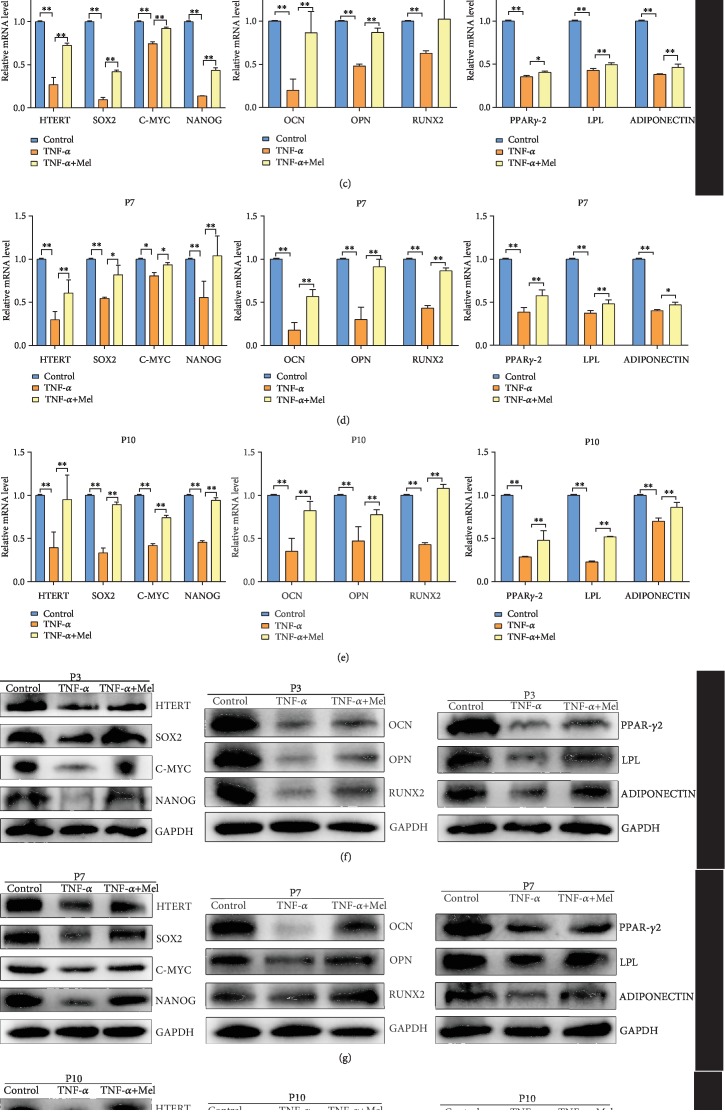
Melatonin effects on HMSC stemness following inflammatory treatment. (a) RT-PCR analysis of biomarkers of HMSC stemness with different concentrations of TNF-*α*. (b) Colony formation assays and quantitative analysis of HMSCs with different treatments. (c) RT-PCR analysis of biomarkers of HMSC stemness, osteogenic differentiation, and adipose differentiation of P3 HMSCs with different treatments. (d) RT-PCR analysis of biomarkers of HMSC stemness, osteogenic differentiation, and adipose differentiation of P7 HMSCs with different treatments. (e) RT-PCR analysis of biomarkers of HMSC stemness, osteogenic differentiation, and adipose differentiation of P10 HMSCs with different treatments. (f) Western blot analysis of biomarkers of HMSC stemness, osteogenic differentiation, and adipose differentiation of P3 HMSCs with different treatments. (g) Western blot analysis of biomarkers of HMSC stemness, osteogenic differentiation, and adipose differentiation of P7 HMSCs with different treatments. (h) Western blot analysis of biomarkers of HMSC stemness, osteogenic differentiation, and adipose differentiation of P10 HMSCs with different treatments. (i) Alizarin red staining and quantification of osteogenic differentiation of HMSCs with different treatments. P3: primary cells expanded to the third generation; P7: primary cells expanded to the seventh generation; P10: primary cells expanded to the tenth generation. ^∗^*P* < 0.05, ^∗∗^*P* < 0.01.

In this study, we employed three different concentrations of TNF-*α* (10 ng/mL, 20 ng/mL, and 50 ng/mL) to simulate an inflammatory environment. RT-PCR analysis of biomarkers of BMMSC stemness (*SOX2*, *C-MYC*, and *NANOG*) showed that mRNA abundances of these biomarkers were significantly lower in the three treated groups than in the control group (*P* < 0.01) (Figure 2(a)). Based on the relative mRNA abundances of these biomarkers in the treatment groups, 20 ng/mL was selected for further analysis. We also evaluated the effects of melatonin on TNF-*α*-treated BMMSCs. For colony formation assays, we selected the P3 generation of BMMSCs. The colony formation ratio in the TNF-*α*-treated group was significantly lower than that in the control (*P* < 0.01). However, colony formation in the TNF-*α*-treated group could be partially recovered with melatonin treatment (*P* < 0.01 vs. TNF-*α*-treated group) (Figure 2(b)). mRNA abundances of biomarkers of BMMSC stemness (*HTERT*, *SOX2*, *C-MYC*, and *NANOG*), osteogenic differentiation (*RUNX2*, *OPN*, and *OCN*), and adipose differentiation (*PPARγ-2*, *LPL*, and *ADIPONECTIN*) in P3, P7, and P10 cells with different treatments were examined with RT-PCR (Figures 2(c)–2(e)). The results showed that the expression levels of *HTERT*, *SOX2*, *C-MYC*, *NANOG*, *RUNX2*, *OPN*, *OCN*, *PPARγ-2*, *LPL*, and ADIPONECTIN in TNF-*α*-treated P3, P7, and P10 cells were significantly lower than those in the control group (*P* < 0.01). However, the expression levels of these genes were recovered with additional melatonin treatment (*P* < 0.01 vs. TNF-*α*-treated group). Similar results were found in western blot analysis. Protein abundances of these biomarkers in TNF-*α*-treated P3, P7, and P10 cells were also significantly lower than those in the control group (*P* < 0.01). Meanwhile, melatonin treatment could partially recover the protein expression of these genes (*P* < 0.01) (Figures 2(f)–2(h)). Alizarin red staining was used to investigate osteogenic differentiation of BMMSCs in P3 cells. Quantification revealed that the OD in the TNF-*α*-treated group was significantly lower than that in the control group (*P* < 0.01). Melatonin treatment was able to increase mineral deposition in the TNF-*α*-treated group (*P* < 0.01 vs. TNF-*α*-treated group) (Figure 2(i)). Therefore, melatonin was effective at reversing the loss of BMMSC stemness caused by inflammatory environment.

### 3.3. Melatonin Reverses the Inflammatory-Induced Loss of BMMSC Stemness via Its Receptor

**Figure 3:**
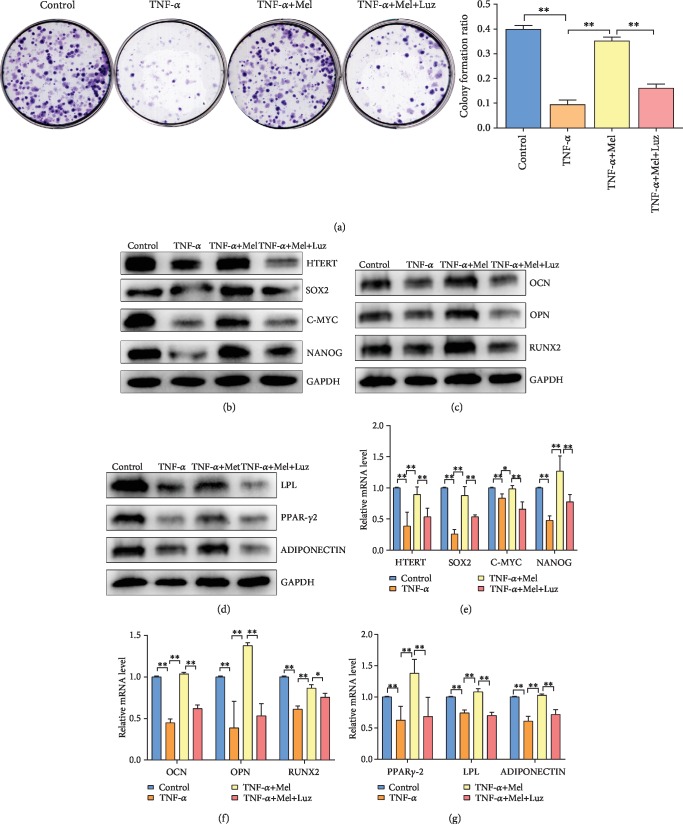
Melatonin reverses HMSC stemness via melatonin receptor. (a) Colony formation assay and quantitative analysis of P3 HMSCs with different treatments. (b) Western blot analysis of biomarkers of HMSC stemness of P3 HMSCs with different treatments. (c) Western blot analysis of biomarkers of osteogenic differentiation of P3 HMSCs with different treatments. (d) Western blot analysis of biomarkers of adipose differentiation of P3 HMSCs with different treatments. (e) RT-PCR analysis of biomarkers of HMSC stemness of P3 HMSCs with different treatments. (f) RT-PCR analysis of biomarkers of osteogenic differentiation of P3 HMSCs with different treatments. (g) RT-PCR analysis of biomarkers of adipose differentiation of P3 HMSCs with different treatments. P3: primary cells expanded to the third generation; ^∗^*P* < 0.05, ^∗∗^*P* < 0.01.

In this study, we have employed luzindole (a melatonin receptor inhibitor) to study the molecular mechanism of melatonin in BMMSC P3 generation. BMMSCs were divided into four groups: control, TNF-*α*, TNF-*α*+Mel, and TNF-*α*+Mel+Luz. For colony formation analyses, colony formation ratio in the TNF-*α* group was significantly lower than that in the control (*P* < 0.01). Colony formation ratio in the TNF-*α*+Mel group was significantly higher than that in the TNF-*α* group (*P* < 0.01). However, this value in the TNF-*α*+Mel+Luz group was significantly lower than that in the TNF-*α*+Mel group (*P* < 0.01) (Figure 3(a)). RT-PCR and western blot analyses of BMMSC stemness (HTERT, SOX2, C-MYC, and NANOG levels), osteogenic differentiation (RUNX2, OPN, and OCN levels), and adipose differentiation (PPAR*γ*-2, LPL, and ADIPONECTIN levels) in P3 cells with different treatments were examined (Figures 3(b)–3(g)). The results show that the mRNA and protein expression levels in BMMSC P3 cells of these molecular mechanisms in the TNF-*α*+Mel+Luz group were significantly lower than those in the TNF-*α*+Mel group (*P* < 0.01). Therefore, we speculated that melatonin could reverse the inflammatory effects on BMMSC stemness via its receptor.

### 3.4. Melatonin Reverses the Inflammation-Induced Loss of BMMSC Stemness via YAP Gene Expression

**Figure 4:**
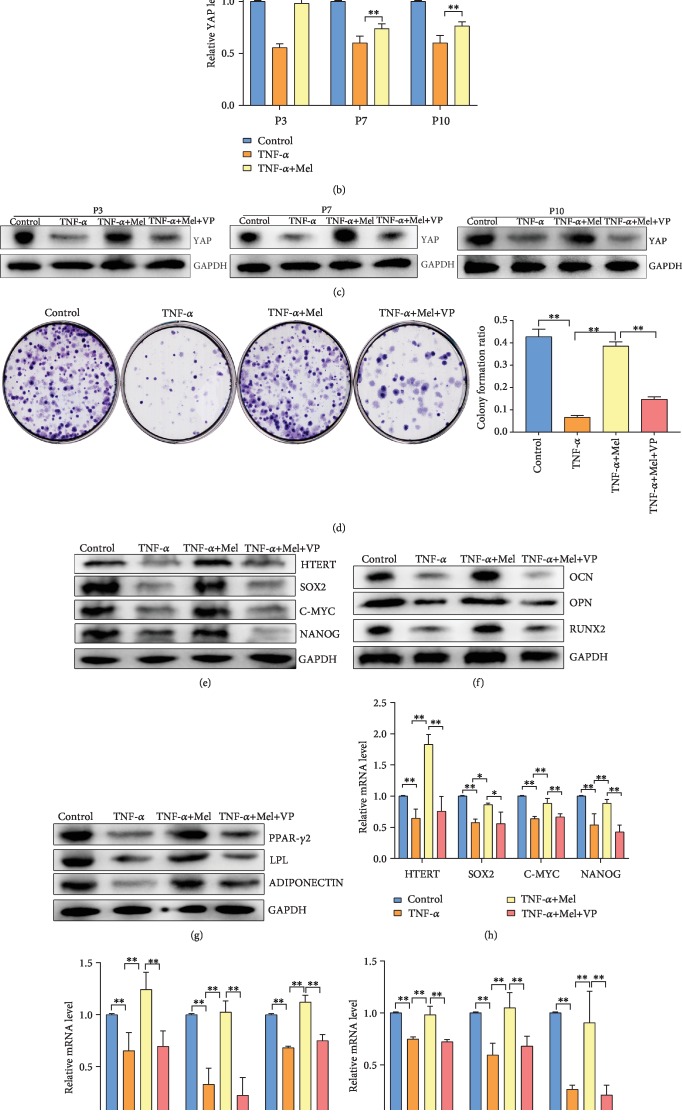
Melatonin reverses loss of HMSC stemness via YAP gene expression. (a) Western blot analysis of YAP protein in P3, P7, and P10 HMSCs with different treatments. (b) RT-PCR analysis of YAP mRNA in P3, P7, and P10 HMSCs with different treatments. (c) Western blot analysis of YAP protein in P3, P7, and P10 HMSCs with different treatments. (d) Colony formation assays and quantitative analysis of P3 HMSCs with different treatments. (e) Western blot analysis of biomarkers of HMSC stemness of P3 HMSCs with different treatments. (f) Western blot analysis of biomarkers of osteogenic differentiation of P3 HMSCs with different treatments. (g) Western blot analysis of biomarkers of adipose differentiation of P3 HMSCs with different treatments. (h) RT-PCR analysis of biomarkers of HMSC stemness of P3 HMSCs with different treatments. (i) RT-PCR analysis of biomarkers of osteogenic differentiation of P3 HMSCs with different treatments. (j) RT-PCR analysis of biomarkers of adipose differentiation of P3 HMSCs with different treatments. P3: primary cells expanded to the third generation; P7: primary cells expanded to the seventh generation; P10: primary cells expanded to the tenth generation. ^∗^*P* < 0.05, ^∗∗^*P* < 0.01.

In this study, we have employed 5 *μ*M verteporfin (a YAP inhibitor) to study the molecular mechanisms of melatonin on BMMSC stemness. BMMSCs were divided into four groups: control, TNF-*α*, TNF-*α*+Mel, and TNF-*α*+Mel+VP. RT-PCR and western blot analyses of YAP expression showed that TNF-*α* treatment could significantly decrease YAP mRNA and protein abundance in P3, P7, and P10 BMMSCs. However, melatonin treatment could reverse YAP mRNA and protein expression in TNF-*α*-treated BMMSCs (*P* < 0.01) (Figures 4(a) and 4(b)). Moreover, Figure 4(c) shows that YAP protein expression in the TNF-*α*+Mel+VP group was significantly lower than that in the TNF-*α*+Mel group in P3, P7, and P10 cells (*P* < 0.01) (Figure 4(c)). For colony formation analyses, colony formation ratio in the TNF-*α*+Mel+VP group was significantly lower than that in the TNF-*α*+Mel-treated group (*P* < 0.01) (Figure 4(d)). In addition, a similar situation was observed in western blot and RT-PCR analyses of BMMSC stemness (HTERT, SOX2, C-MYC, and NANOG levels), osteogenic differentiation (RUNX2, OPN, and OCN levels), and adipose differentiation (PPAR*γ*-2, LPL, and ADIPONECTIN levels) in P3 cells with different treatments (Figures 4(e)–4(j)). The mRNA and protein expression levels of these genes in the TNF-*α*+Mel+VP group was significantly lower than those in the TNF-*α*+Mel group (*P* < 0.01). In summary, YAP is a potential target of melatonin in reversing the impacts of inflammation on BMMSC stemness.

### 3.5. BMMSC Treatment in an Osteoporosis Nude Mouse Model

**Figure 5:**
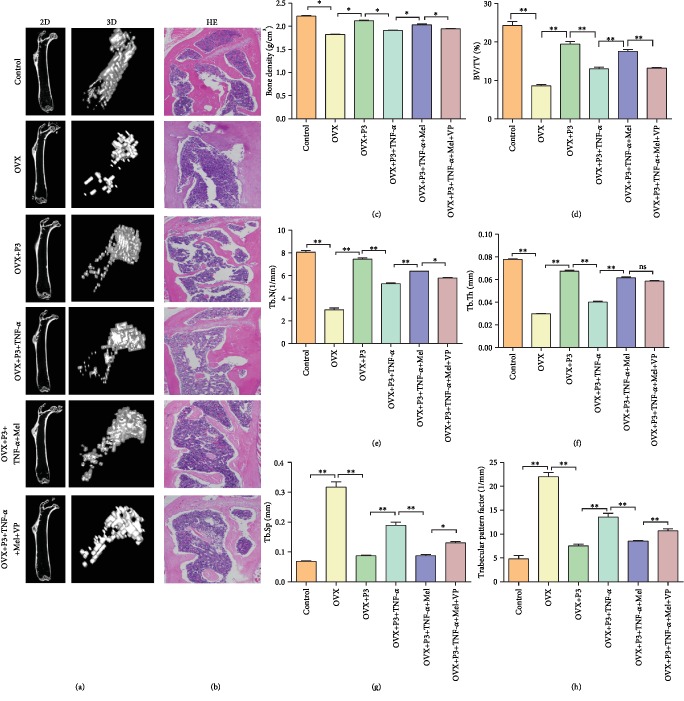
Stem cell treatment of osteoporosis mouse model. (a) Micro-CT examination of two-dimensional and three-dimensional map of trabecular bone in different groups. (b) H&E staining of the proximal femur of mice in different groups. (c) Bone density analysis of osteoporosis mouse with different treatments. (d) Bone volume density (BV/TV) of osteoporosis mouse with different treatments. (e) Trabecular number (Tb.N) of osteoporosis mouse with different treatments. (f) Trabecular thickness (Tb.Th) of osteoporosis mouse with different treatments. (g) Trabecular separation (Tb.Sp) of osteoporosis mouse with different treatments. (h) Trabecular pattern factor of osteoporosis mouse with different treatments. Ten nude mice were included in each group. ^∗^*P* < 0.05, ^∗∗^*P* < 0.01.

In this study, we constructed an osteoporosis mouse model to study the effects and potential molecular mechanisms of BMMSC treatment.  Figure 5(a) shows the results of micro-CT of two-dimensional (2D) and three-dimensional (3D) map of trabecular bone in different groups. The results suggest that construction of the osteoporosis mouse model was successful (via OVX treatment). BMMSC treatment could partially recovery the damage caused by osteoporosis (OVX+P3 BMSC treatment). TNF-*α* treatment aggravated osteoporosis. However, melatonin was able to reverse the damage of osteoporosis. VP, one of the YAP inhibitors, was shown to weaken the therapeutic effect of melatonin in osteoporosis. Similar results were also observed in H&E staining analysis (Figure 5(b)). Quantitative analysis of osteoporosis-related indices suggested that the values of bone density, BV/TV, Tb.N, and Tb.Th were significantly different in the six subgroups. For example, the above indices in the OVX group were significantly lower than those in the control group (*P* < 0.05). Meanwhile, the above indices in the OVX+P3 BMSC+TNF-*α*+Mel group were significantly higher than those in the OVX+P3 BMSC+TNF-*α* group (*P* < 0.05). However, VP could reverse the expression in the OVX+P3 BMSC+TNF-*α*+Mel+VP group (Figures 5(c)–5(f)). In addition, Tb.Sp and trabecular pattern factor analysis indicated that both indices in different groups were also significantly different in the six subgroups. For example, both indices in the OVX group were significantly higher than those in the control group (*P* < 0.01). Meanwhile, both indices in the OVX+P3 BMSC+TNF-*α*+Mel group were significantly lower than those in the OVX+P3 BMSC+TNF-*α* group (*P* < 0.01). However, they were upregulated in the OVX+P3 BMSC+TNF-*α*+Mel+VP group compared with the OVX+P3 BMSC+TNF-*α*+Mel group (Figures 5(g) and 5(h)).

## 4. Discussion

Stemness is the ability of stem cells to maintain pluripotent differentiation potential and undergo no directional differentiation [29]. In the early stage of *in vitro* stem cell culture, the stemness of stem cells can be maintained. However, the stemness of stem cells decreases with an extension of culture time [30]. Meanwhile, when *in vitro*-cultured stem cells are injected into the body, the stemness of the MSCs is also reduced [31, 32]. Due to the reduction in stemness, MSCs reduce tissue damage mainly through anti-inflammatory effects rather than by differentiating into specific tissue types in tissue damage treatments following myocardial infarction and spinal cord injury [33, 34]. The maintenance of MSC stemness is related to multiple factors, including aging, *in vitro* generation, and origination. For aging, there are various features correlated with stemness reduction, including adipose tissue accumulation in the bone marrow and reduced ability of osteogenic differentiation [35]. Nicotinamide phosphoribosyltransferase (Nampt) has been shown to affect the balance between MSC adipogenesis and osteogenesis by regulating the activity of the silencing information regulator 1 (sirtl) gene. For *in vitro* generation and origination, the stemness of MSCs can be maintained in the early and middle generations of *in vitro* growth. However, stemness can experience a significant reduction once a critical time is exceeded [36, 37]. Meanwhile, stem cells from different tissue sources have significant differences in their abilities to maintain stemness during *in vitro* culture. For example, the stemness of BMMSCs can be maintained until the 15^th^ generation *in vitro*. However, the stemness then decreases with subsequent culture. Only a very small number of pluripotent cells can be maintained until the 20^th^ generation *in vitro* [38]. Umbilical-derived MSCs showed stable stemness in the early and middle generations of culture; however, markers related to aging, such as CD105 and CD73, were significantly increased after the 21^st^ generation [39]. In our study, with the passage of BMMSCs, their stemness and osteogenic and adipogenic differentiation abilities gradually decreased with increasing generations. Therefore, it is important to achieve a detailed molecular understanding of the mechanisms underlying the maintenance of stemness in MSCs.

TNF-*α* was discovered in 1975 [40], and there are two main members of the TNF family, namely, TNF-*α* and TNF-*β*. TNF-*α* is mainly produced by activated mononuclear macrophages and is closely related to osteoporosis, as high TNF-*α* expression can be detected in cases of osteoporosis [41, 42]. This indicates that TNF-*α* is an important factor in diseases of bone metabolism. TNF-*α* is a trimeric inflammatory factor that binds to transmembrane receptors. TNF-*α* synergizes with a variety of cells to produce a concerted response to inflammatory products, bacterial toxins, and other aggressive stimuli [43]. BMMSCs undergo apoptosis in an inflammatory environment. TNF-*α* can promote apoptosis indirectly through NO regulation [44]. Meanwhile, TNF-*α* inhibits the expression of two transcription factors in HMSCs: Ostrix and Runx2. Therefore, TNF-*α* can reduce the expression of specific markers of osteoblast differentiation, which ultimately inhibits the differentiation of terminal osteoblasts [45]. Moreover, TNF-*α* can regulate the expression of BMP-2 and POEM, thereby inhibiting the differentiation of osteoblasts [46]. In addition, TNF-*α* has a significant effect on the absorption of osteoclast bone [47]. Overall, TNF-*α* can inhibit bone remodeling and promote bone resorption, which results in a reduction of bone mass. In this study, 20 ng/mL TNF-*α* could effectively induce an inflammatory environment in BMMSCs.

Treatment with melatonin can significantly improve the viability of MSCs and stimulate angiogenesis, renal cell proliferation, and renal resuscitation [48]. *In vitro* cell assays have shown that melatonin can induce the production of antioxidant enzymes such as catalase and superoxide dismutase, which can reduce apoptosis of MSCs caused by oxidative stress [49]. Melatonin can also reduce the inflammation of MSCs induced by interleukin-1. Melatonin treatment can increase the mRNA expression of Cu/Zn superoxide dismutase (Cu/ZnSOD) and MnSOD and decrease the expression level of the apoptosis-related Bax gene. The melatonin receptor inhibitor luzindole could inhibit the effects cause by melatonin [50]. In this study, our results show that melatonin treatment in BMMSCs can effectively recover the loss of cell stemness caused by TNF-*α* treatment, which is in agreement with a previous study. Therefore, melatonin treatment was an effective means to rescue BMMSC stemness.

Maintenance of stemness in MSCs is regulated by a variety of cellular signals, such as BMP signaling and Wnt signaling, which can promote the differentiation of MSCs into osteoblasts. However, a lack of BMP signal or Wnt signal will inhibit the differentiation of MSCs into osteoblasts and promote the differentiation of MSCs into fat cells [51–53]. Platelet-derived growth factor BB (PDGF-BB), hepatocyte growth factor, transforming growth factor beta (TGF-*β*), and basic fibroblast growth factor (bFGF) signals can promote the differentiation of MSCs into muscle [54–56]. Recent studies have found that Hippo signaling also plays a key role in MSC fate determination. Hippo signaling is highly conserved in animals and mainly functions to regulate stem cell self-renewal, tissue regeneration, and organ size [57, 58]. YAP is a key transcriptional factor that is negatively regulated by the Hippo pathway, which is a conserved pathway that regulates organ size and tumorigenesis [59, 60]. Recent studies have found that YAP can regulate transcription factors that are crucial for bone homeostasis, such as Runt-related transcription factor 2 (RUNX2) [61], signal transducer and activator of transcription factor 3 (STAT3) [62], and *β*-catenin [63]. RUNX2 is a basic transcription factor that stimulates osteogenesis, and YAP can be used as a coactivator of RUNX2 [61]. Pan et al. reported that YAP could stabilize *β*-catenin and thus increase nuclear *β*-catenin-mediated osteogenesis [63]. In this study, YAP expression was decreased as TNF-*α* inhibited BMMSC stemness. However, melatonin was able to restore YAP expression. Our results confirm that melatonin treatment can reverse the loss of BMMSC stemness caused by TNF-*α* via YAP signaling.

## 5. Conclusions

In summary, we demonstrated that HMSC stemness decreases with increased generations of *in vitro* culture. TNF-*α* could result in the inflammatory-based reduction in HMSC stemness. However, melatonin could reverse the inflammatory reduction of HMSC stemness via the melatonin receptor and YAP signaling. In osteoporotic nude mice, melatonin improved the effects of HMSC treatment. This work provides a detailed understanding of the effects of melatonin treatment of HMSCs.

## Figures and Tables

**Table 1:** Sequences of primers used for quantitative RT-PCR assay.

Gene	Primer sequence (5′-3′)
*GAPDH*	Forward: AGAAAAACCTGCCAAATATGATGAC Reverse: TGGGTGTCGCTGTTGAAGTC
*RUNX2*	Forward: AGAAGGCACAGACAGAAGCTTGA Reverse: AGGAATGCGCCCTAAATCACT
*OPN*	Forward: GCGAGGAGTTGAATGGTG Reverse: CTTGTGCTGTGGGTTTC
*OCN*	Forward: CACTCCTCGCCCTATTGGC Reverse: CCCTCCTGCTTGGACACAAAG
*HTERT*	Forward: AAATGCGGCCCCTGTTTCT Reverse: CAGTGCGTCTTGAGGAGCA
*SOX2*	Forward: GCCGAGTGGAAACTTTTGTCG Reverse: GGCAGCGTGTACTTATCCTTCT
*C-MYC*	Forward: GGCTCCTGGCAAAAGGTCA Reverse: CTGCGTAGTTGTGCTGATGT
*NANOG*	Forward: TTTGTGGGCCTGAAGAAAACT Reverse: AGGGCTGTCCTGAATAAGCAG
*PPARγ-2*	Forward: ACCAAAGTGCAATCAAAGTGGA Reverse: ATGAGGGAGTTGGAAGGCTCT
*LPL*	Forward: ACAAGAGAGAACCAGACTCCAA Reverse: GCGGACACTGGGTAATGCT
*ADIPONECTIN*	Forward: GGCTTTCCGGGAATCCAAGG Reverse: TGGGGATAGTAACGTAAGTCTCC
*YAP*	Forward: TAGCCCTGCGTAGCCAGTTA Reverse: TCATGCTTAGTCCACTGTCTGT

## Data Availability

The datasets analyzed during the current study are available from the corresponding authors on reasonable request.

## Abbreviations

MSCs: Mesenchymal stem cells
BMMSCs: Bone marrow mesenchymal stem cells
HMSCs: Human bone marrow mesenchymal stem cells
BMP: Bone morphogenetic protein
HTERT: Human telomerase reverse transcriptase
OPN: Osteopontin
OCN: Osteocalcin
RUNX2: Recombinant runt-related transcription factor 2
PPAR*γ*-2: Peroxisome proliferator-activated receptor-*γ*
LPL: Lipoprotein lipase
H&E: Hematoxylin and eosin staining.

## Supplementary Materials

Supplementary Materials Supplementary Figure 1: identification of isolated BMMSCs. Flow cytometric analysis of CD34 (a), CD45 (b), CD73 (c), CD90 (d), and CD105 (e).

## References

[1] Charbord P. (2010). Bone marrow mesenchymal stem cells: historical overview and concepts. *Human Gene Therapy*.

[2] Han S., Zhao Y., Xiao Z. (2012). The three-dimensional collagen scaffold improves the stemness of rat bone marrow mesenchymal stem cells. *Journal of Genetics and Genomics*.

[3] Zhang Y., Khan D., Delling J., Tobiasch E. (2012). Mechanisms underlying the osteo- and adipo-differentiation of human mesenchymal stem cells. *The Scientific World Journal*.

[4] Nancarrow-Lei R., Mafi P., Mafi R., Khan W. (2017). A systemic review of adult mesenchymal stem cell sources and their multilineage differentiation potential relevant to musculoskeletal tissue repair and regeneration. *Current Stem Cell Research & Therapy*.

[5] Brandl A., Meyer M., Bechmann V., Nerlich M., Angele P. (2011). Oxidative stress induces senescence in human mesenchymal stem cells. *Experimental Cell Research*.

[6] Kassem M., Marie P. J. (2011). Senescence-associated intrinsic mechanisms of osteoblast dysfunctions. *Aging Cell*.

[7] Li J., Pei M. (2012). Cell senescence: a challenge in cartilage engineering and regeneration. *Tissue Engineering Part B: Reviews*.

[8] Jacobsen S. E. W. (2005). Defining 'stemness': Notch and Wnt join forces?. *Nature Immunology*.

[9] Luo K. (2017). Signaling cross talk between TGF-*β*/Smad and other signaling pathways. *Cold Spring Harbor Perspectives in Biology*.

[10] Kyurkchiev D., Bochev I., Ivanova-Todorova E. (2014). Secretion of immunoregulatory cytokines by mesenchymal stem cells. *World Journal of Stem Cells*.

[11] Zhao B. (2017). TNF and bone remodeling. *Current Osteoporosis Reports*.

[12] Qiu X., Wang X., Qiu J. (2019). Melatonin rescued reactive oxygen species-impaired osteogenesis of human bone marrow mesenchymal stem cells in the presence of tumor necrosis factor-alpha. *Stem Cells International*.

[13] Zisapel N. (2018). New perspectives on the role of melatonin in human sleep, circadian rhythms and their regulation. *British Journal of Pharmacology*.

[14] Galano A., Tan D. X., Reiter R. J. (2013). On the free radical scavenging activities of melatonin's metabolites, AFMK and AMK. *Journal of Pineal Research*.

[15] Zhang H. M., Zhang Y. (2014). Melatonin: a well-documented antioxidant with conditional pro-oxidant actions. *Journal of Pineal Research*.

[16] Liu X., Xu Y., Chen S. (2014). Rescue of proinflammatory cytokine-inhibited chondrogenesis by the antiarthritic effect of melatonin in synovium mesenchymal stem cells via suppression of reactive oxygen species and matrix metalloproteinases. *Free Radical Biology and Medicine*.

[17] Tan D.-x., Manchester L. C., Reiter R. J. (1999). Identification of highly elevated levels of melatonin in bone marrow: its origin and significance. *Biochimica et Biophysica Acta (BBA)-General Subjects*.

[18] Luchetti F., Canonico B., Bartolini D. (2014). Melatonin regulates mesenchymal stem cell differentiation: a review. *Journal of Pineal Research*.

[19] Radio N. M., Doctor J. S., Witt-Enderby P. A. (2006). Melatonin enhances alkaline phosphatase activity in differentiating human adult mesenchymal stem cells grown in osteogenic medium via MT2 melatonin receptors and the MEK/ERK (1/2) signaling cascade. *Journal of Pineal Research*.

[20] Yagi R., Chen L. F., Shigesada K., Murakami Y., Ito Y. (1999). A WW domain-containing yes-associated protein (YAP) is a novel transcriptional co-activator. *The EMBO Journal*.

[21] Zhang L., Su P., Xu C. (2010). Melatonin inhibits adipogenesis and enhances osteogenesis of human mesenchymal stem cells by suppressing PPAR*γ* expression and enhancing Runx2 expression. *Journal of Pineal Research*.

[22] Zhang L., Zhang J., Ling Y. (2013). Sustained release of melatonin from poly (lactic-co-glycolic acid)(PLGA) microspheres to induce osteogenesis of human mesenchymal stem cells in vitro. *Journal of Pineal Research*.

[23] Lian C., Wu Z., Gao B. (2016). Melatonin reversed tumor necrosis factor-alpha-inhibited osteogenesis of human mesenchymal stem cells by stabilizing SMAD 1 protein. *Journal of Pineal Research*.

[24] Ma X., Xu Z., Ding S., Yi G., Wang Q. (2017). Alendronate promotes osteoblast differentiation and bone formation in ovariectomy-induced osteoporosis through interferon-*β*/signal transducer and activator of transcription 1 pathway. *Experimental and Therapeutic Medicine*.

[25] Chan J. K. C. (2014). The wonderful colors of the hematoxylin–eosin stain in diagnostic surgical pathology. *International Journal of Surgical Pathology*.

[26] Zhang Z.-M., Li Z.-C., Jiang L.-S., Jiang S.-D., Dai L.-Y. (2010). Micro-CT and mechanical evaluation of subchondral trabecular bone structure between postmenopausal women with osteoarthritis and osteoporosis. *Osteoporosis International*.

[27] Gong H., Zhang M., Yeung H. Y., Qin L. (2005). Regional variations in microstructural properties of vertebral trabeculae with aging. *Journal of Bone and Mineral Metabolism*.

[28] Hildebrand T., Laib A., Müller R., Dequeker J., Rüegsegger P. (1999). Direct three-dimensional morphometric analysis of human cancellous bone: microstructural data from spine, femur, iliac crest, and calcaneus. *Journal of Bone and Mineral Research*.

[29] Guo X., Tang Y., Zhang P. (2019). Effect of ectopic high expression of transcription factor OCT4 on the “stemness” characteristics of human bone marrow-derived mesenchymal stromal cells. *Stem Cell Research & Therapy*.

[30] Jiang T., Xu G., Wang Q. (2017). *In vitro* expansion impaired the stemness of early passage mesenchymal stem cells for treatment of cartilage defects. *Cell Death & Disease*.

[31] Chen J., Sanberg P. R., Li Y. (2001). Intravenous administration of human umbilical cord blood reduces behavioral deficits after stroke in rats. *Stroke*.

[32] Hicks A. U., Lappalainen R. S., Narkilahti S. (2009). Transplantation of human embryonic stem cell-derived neural precursor cells and enriched environment after cortical stroke in rats: cell survival and functional recovery. *European Journal of Neuroscience*.

[33] Hu J., Yan Q., Shi C., Tian Y., Cao P., Yuan W. (2017). BMSC paracrine activity attenuates interleukin-1*β*-induced inflammation and apoptosis in rat AF cells via inhibiting relative NF-*κ*B signaling and the mitochondrial pathway. *American Journal of Translational Research*.

[34] Han D., Wu C., Xiong Q., Zhou L., Tian Y. (2015). Anti-inflammatory mechanism of bone marrow mesenchymal stem cell transplantation in rat model of spinal cord injury. *Cell Biochemistry and Biophysics*.

[35] Chen Q., Shou P., Zheng C. (2016). Fate decision of mesenchymal stem cells: adipocytes or osteoblasts?. *Cell Death and Differentiation*.

[36] Wang H., Hu Z., Wu J. (2019). Sirt1 promotes osteogenic differentiation and increases alveolar bone mass via Bmi1 activation in mice. *Journal of Bone and Mineral Research*.

[37] Ma C., Pi C., Yang Y. (2017). Nampt expression decreases age-related senescence in rat bone marrow mesenchymal stem cells by targeting Sirt1. *PloS One*.

[38] Shuai Y., Liao L., Su X. (2016). Melatonin treatment improves mesenchymal stem cells therapy by preserving stemness during long-term in vitro expansion. *Theranostics*.

[39] Arutyunyan I., Elchaninov A., Makarov A., Fatkhudinov T. (2016). Umbilical cord as prospective source for mesenchymal stem cell-based therapy. *Stem Cells International*.

[40] Parameswaran N., Patial S. (2010). Tumor necrosis factor-*α* signaling in macrophages. *Critical Reviews in Eukaryotic Gene Expression*.

[41] Iseme R. A., Mcevoy M., Kelly B., Agnew L., Walker F. R., Attia J. (2017). Is osteoporosis an autoimmune mediated disorder?. *Bone Reports*.

[42] Amarasekara D. S., Yu J., Rho J. (2015). Bone loss triggered by the cytokine network in inflammatory autoimmune diseases. *Journal of Immunology Research*.

[43] Chen L., Deng H., Cui H. (2018). Inflammatory responses and inflammation-associated diseases in organs. *Oncotarget*.

[44] Kim J. J., Lee S. B., Park J. K., Yoo Y. D. (2010). TNF- *α* -induced ROS production triggering apoptosis is directly linked to Romo1 and Bcl-X_L_. *Cell Death and Differentiation*.

[45] Gilbert L., He X., Farmer P. (2000). Inhibition of osteoblast differentiation by tumor necrosis factor-*α*. *Endocrinology*.

[46] Tsukasaki M., Yamada A., Suzuki D. (2011). Expression of POEM, a positive regulator of osteoblast differentiation, is suppressed by TNF-*α*. *Biochemical and Biophysical Research Communications*.

[47] Bi H., Chen X., Gao S. (2017). Key triggers of osteoclast-related diseases and available strategies for targeted therapies: a review. *Frontiers in Medicine*.

[48] de Cássia Noronha N., Mizukami A., Caliári-Oliveira C. (2019). Priming approaches to improve the efficacy of mesenchymal stromal cell-based therapies. *Stem Cell Research & Therapy*.

[49] Chang C.-C., Huang T.-Y., Chen H.-Y. (2018). Protective effect of melatonin against oxidative stress-induced apoptosis and enhanced autophagy in human retinal pigment epithelium cells. *Oxidative Medicine and Cellular Longevity*.

[50] Hu C., Li L. (2019). Melatonin plays critical role in mesenchymal stem cell-based regenerative medicine in vitro and in vivo. *Stem Cell Research & Therapy*.

[51] Caplan A. I. (1991). Mesenchymal stem cells. *Journal of Orthopaedic Research*.

[52] Baksh D., Boland G. M., Tuan R. S. (2007). Cross-talk between Wnt signaling pathways in human mesenchymal stem cells leads to functional antagonism during osteogenic differentiation. *Journal of Cellular Biochemistry*.

[53] McBeath R., Pirone D. M., Nelson C. M., Bhadriraju K., Chen C. S. (2004). Cell shape, cytoskeletal tension, and RhoA regulate stem cell lineage commitment. *Developmental Cell*.

[54] Wu R., Liu G., Bharadwaj S., Zhang Y. (2013). Isolation and myogenic differentiation of mesenchymal stem cells for urologic tissue engineering. *Organ Regeneration*.

[55] Beier J. P., Bitto F. F., Lange C. (2011). Myogenic differentiation of mesenchymal stem cells co-cultured with primary myoblasts. *Cell Biology International*.

[56] Lee K.-D. (2008). Applications of mesenchymal stem cells: an updated review. *Chang Gung Medical Journal*.

[57] Ramos A., Camargo F. D. (2012). The Hippo signaling pathway and stem cell biology. *Trends in Cell Biology*.

[58] Zhao B., Tumaneng K., Guan K.-L. (2011). The Hippo pathway in organ size control, tissue regeneration and stem cell self-renewal. *Nature Cell Biology*.

[59] Yu J., Zheng Y., Dong J., Klusza S., Deng W.-M., Pan D. (2010). Kibra functions as a tumor suppressor protein that regulates Hippo signaling in conjunction with Merlin and Expanded. *Developmental Cell*.

[60] Lei Q.-Y., Zhang H., Zhao B. (2008). TAZ promotes cell proliferation and epithelial-mesenchymal transition and is inhibited by the hippo pathway. *Molecular and Cellular Biology*.

[61] Zaidi S. K., Sullivan A. J., Medina R. (2004). Tyrosine phosphorylation controls Runx2-mediated subnuclear targeting of YAP to repress transcription. *The Embo Journal*.

[62] Huang Z., Wang Y., Hu G., Zhou J., Mei L., Xiong W.-C. (2016). YAP is a critical inducer of SOCS3, preventing reactive astrogliosis. *Cerebral Cortex*.

[63] Pan J.-X., Xiong L., Zhao K. (2018). YAP promotes osteogenesis and suppresses adipogenic differentiation by regulating *β*-catenin signaling. *Bone Research*.

