# Corrigendum to “Melatonin Reverses the Loss of Stemness Induced by TNF-*α* in Human Bone Marrow Mesenchymal Stem Cells through Upregulation of YAP Expression”

**DOI:** 10.1155/2022/9823765

**Published:** 2022-01-20

**Authors:** Xudong Wang, Tongzhou Liang, Jincheng Qiu, Xianjian Qiu, Bo Gao, Wenjie Gao, Chengjie Lian, Taiqiu Chen, Yuanxin Zhu, Anjing Liang, Peiqiang Su, Yan Peng, Dongsheng Huang

**Affiliations:** ^1^Department of Orthopedics, Sun Yat-sen Memorial Hospital of Sun Yat-sen University, Guangzhou, Guangdong 510120, China; ^2^Department of Orthopedics, The First Affiliated Hospital of Sun Yat-sen University, Guangzhou, Guangdong 510080, China

In the article titled “Melatonin Reverses the Loss of Stemness Induced by TNF-*α* in Human Bone Marrow Mesenchymal Stem Cells through Upregulation of YAP Expression” [1], there was an error in Figure 5. The authors apologize for this error and confirm that it does not affect the results and the conclusions of the article. The corrected figure, as approved by the editorial board is as follows:

## Figures and Tables

**Figure 1:**
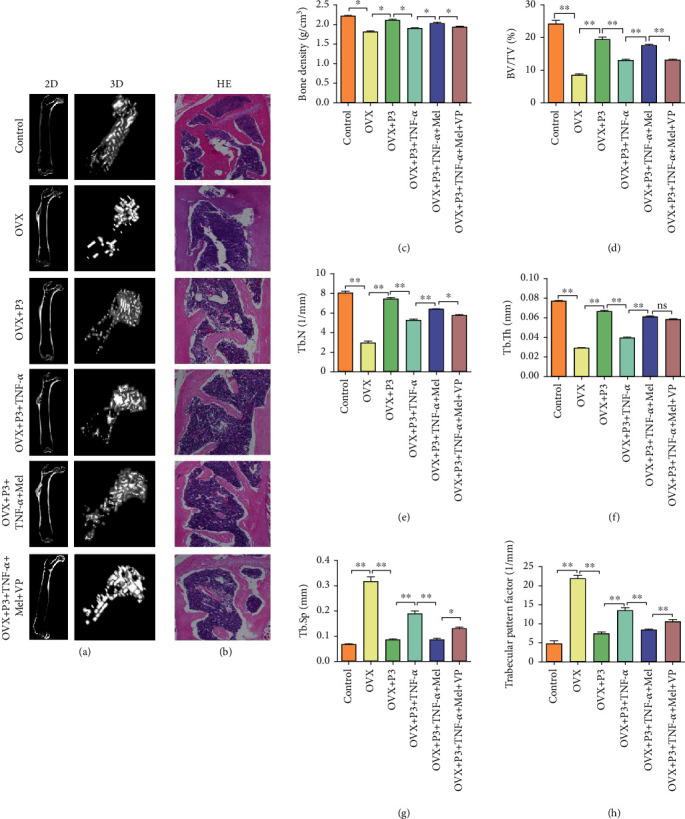
Stem cell treatment of osteoporosis mouse model. (a) Micro-CT examination of a two-dimensional and three-dimensional map of trabecular bone in different groups. (b) H&E staining of the proximal femur of mice in different groups. (c) Bone density analysis of osteoporosis mouse with different treatments. (d) Bone volume density (BV/TV) of osteoporosis mouse with different treatments. (e) Trabecular number (Tb.N) of osteoporosis mouse with different treatments. (f) Trabecular thickness (Tb.Th) of osteoporosis mouse with different treatments. (g) Trabecular separation (Tb.Sp) of osteoporosis mouse with different treatments. (h) Trabecular pattern factor of osteoporosis mouse with different treatments. Ten nude mice were included in each group. ^∗^*P* < 0.05; ^∗∗^*P* < 0.01.
