# Supplementary material for: Melatonin Reverses the Loss of Stemness Induced by TNF-α in Human Bone Marrow Mesenchymal Stem Cells through Upregulation of YAP Expression
Source: Stem Cells Int. 2019 Dec 30;2019:6568394. doi: 10.1155/2019/6568394 (PMC7012241; doi:10.1155/2019/6568394)
Supplement: Supplementary Materials — Supplementary Figure 1: identification of isolated BMMSCs. Flow cytometric analysis of CD34 (a), CD45 (b), CD73 (c), CD90 (d), and CD105 (e). [file 6568394.f1.pdf]

Stem Cells International

Melatonin reverses the loss of stemness induced by TNF-α in human bone marrow mesenchymal stem cells through up-regulation of YAP expression

SUPPLEMENTARY FIGURES AND FIGURE LEGENDS

Supplementary Figure 1

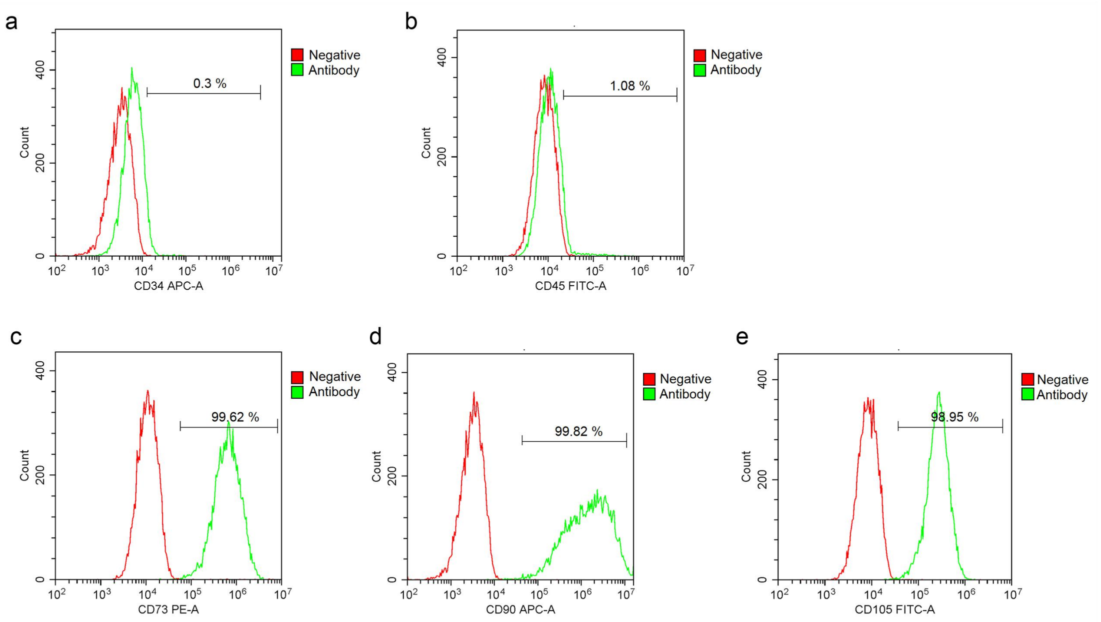

Supplementary Figure 1 Identification of isolated BMMSCs. Flow cytometric analysis of CD34(**a**), CD45(**b**), CD73(**c**), CD90(**d**), and CD105(**e**).
